# Expression of VEGF-A Signaling Pathway in Cartilage of ACLT-induced Osteoarthritis Mouse Model

**DOI:** 10.1186/s13018-021-02528-w

**Published:** 2021-06-14

**Authors:** Jia-jia Qian, Qi Xu, Wei-min Xu, Ren Cai, Gui-cheng Huang

**Affiliations:** 1 Laboratory for New Techniques of Restoration & Reconstruction of Orthopedics and Traumatology, Nanjing University of Chinese Medicine, Nanjing, Jiangsu China; 2 Department of Rehabilitation Therapy, Nanjing University of Chinese Medicine, Nanjing, Jiangsu China; 3 Department of Basic Physical Education, Nanjing University of Chinese Medicine, Nanjing, Jiangsu China

**Keywords:** Osteoarthritis, ACLT, VEGF-A, Angiogenesis, Inflammation

## Abstract

**Background:**

Anterior cruciate ligament transection surgery (ACLT)-induced OA model was often used to investigate the molecular mechanism of knee osteoarthritis (KOA). Researches have shown that vascular endothelial growth factor (VEGF) played an important role in OA. The present study aimed to investigate the pathological changes after ACLT surgery and reveal the expression characteristics of the VEGF-A/VEGFR2 signaling pathway in this model.

**Methods:**

Moderate KOA model was established by ACLT, and 1, 2, 4, 8, and 12 weeks after surgery, hematoxylin-eosin (HE) and Safranin-O(S-O) staining were used to detect the pathological changes in mouse knee cartilage, and the matrix biomarkers A Disintegrin and Metalloproteinase with Thrombospondin Motifs 5(ADAMTS5), Collagen II (COL-II) were detected using immunohistochemistry (IHC), CD31 was detected by immunofluorescence (IF) to show the vascular invasion in cartilage, and proteins expression of VEGF-A pathway were detected by Western blot (WB). Meanwhile, the inflammatory biomarkers cyclooxygenase-2 (COX-2) and inducible nitric oxide synthase (iNOS) in cartilage were detected by WB.

**Results:**

ACLT surgery can lead to degeneration of cartilage in mice, and the characteristics of the lesion were time-dependent. The ADAMTS5-positive cells increased while COL-II decreased in OA cartilage with time, and new blood vessels labeled by CD31 can be seen from 1 week in OA cartilage, and increased in 8 and 12 weeks. The expression of VEGF-A, VEGFR2, COX-2, and iNOS were higher than control groups, which were basically consistent with the degree of osteoarthritis.

**Conclusions:**

The degenerative degree of articular cartilage was time-dependent; angiogenesis and inflammation were important pathological changes of cartilage in KOA. The expression of the VEGF-A/VEGFR2 signaling pathway was basically correlated with the degree of KOA.

## Background

Osteoarthritis (OA) is an irreversible degenerative arthritis disease [1, 2], and OA-associated degeneration of articular cartilages, synovitis, and the formation of osteophyte have become the main reasons underlying adult disability, especially in the elderly, and OA is estimated to be the fourth leading cause of disability by 2020 [3–5]. It is well known that increased age, obesity, joint injuries, and lifestyle are all risk factors for OA [6], and these factors are closely related to the mechanical loading to the joints, so it is assumed that a large part of OA is induced by accumulated mechanical stress [7]. ACLT-induced OA model is a normal stress-induced OA model in mice [7]. As previously reported, the ACLT mouse model demonstrates great similarities with human osteoarthritis, including subchondral change, articular cartilage damage, and synovitis [8–10], which made this model ideal for the research of OA.

Nowadays, although the clear understanding of the underlying mechanism of OA remains elusive [11], researches have shown that angiogenesis and inflammation are important processes in the pathophysiology of OA [12, 13]. Which can cause joint damage, endochondral ossification, and pain. VEGF was a potent stimulator of angiogenesis, which can also contribute to inflammation [14]. Till now, the angiogenesis and inflammation reaction in different pathological stages of the ACLT-induced OA model has never been revealed. Clarifying the correlation of VEGF, angiogenesis, and inflammation reaction in OA pathological process may provide experimental support for OA pathogenesis study.

Therefore, in this study, we established a moderate OA model by ACLT surgery, to explore the histopathological changes, angiogenesis, and inflammation reaction of knee cartilage in different pathological stages. We also tried to explore the expression of the VEGF-A/VEGFR2 signaling pathway in the cartilage of the ACLT model.

## Materials and methods

### Animas models

Eighty-eight C57BL/6 mice (12-week-old) of both sexes were purchased from Shandong skobas Biotechnology Co., Ltd. (Shandong, China). Mice were housed in the Laboratory Animal Center of Nanjing University of Chinese medicine under specific pathogen-free (SPF) and maintained at 27 °C under a 12-h light/dark cycle with 50% of humidity throughout the experiments. Experiments performed in this study were all approved by the Animal Experiment Committee of Nanjing University of Chinese Medicine (ethics No:201904A009).

Eight mice served as control animals (Blank B), and the others were randomly divided into two groups, namely the model group (*n* = 40) and the sham group (*n* = 40). Each group was further divided into five subgroups depending on when the animals were killed (*n* = 8). We established a surgically induced moderate OA model by anterior cruciate ligament transection surgery (ACLT) which was described in previous reseaches [7, 15], and the groups were named as M1, M2, M4, M8, M12 (1, 2, 4, 8, 12 weeks after ACLT). In the sham group, only a 1.5-cm incision in the same position was made without cutting the ligament, and then the groups were named as S1, S2, S4, S8, S12 (1, 2, 4, 8, 12 weeks after sham surgery).

### Hematoxylin–eosin (HE) and Safranin-O (S-O) staining

Knee tissues of each group were fixed in 4% paraformaldehyde for 24 h and then decalcified in 10% EDTA for 8 weeks. After dehydrated in graded ethanol, tissues were embedded in paraffin. Four-micrometer sections were stained with hematoxylin-eosin and Safranin O-Fast Green. Three to five fields were randomly selected from each section and observed under a × 100 light microscope. The pathological changes were evaluated thrice and graded by 3 independent researchers using the Mankin histological criteria, which was scored according to structural integrity, cartilage cells, Safranin-O staining, and tidemark integrity [16].

### Immunofluorescence and immunohistochemistry

Knee joint sections (4 μm) were deparaffinized at 37 °C for 30 min and then hydrated with xylene, graded alcohol; after antigen retrieval was performed, blocked for 10 min with 3% H2O2 methanol solution at room temperature, sections were then incubated with anti CD31(1:500, abcam ab182981), anti-ADAMTS5(1:100, Bioss), and anti COL-II (1:100, Affinity AF0135), incubated overnight at 4 °C; 50 UL of Sheep anti-rabbit polymer was added for 20 min at room temperature;DAB was added for color development hematoxylin counterstaining for 10 min. The protein expression was observed under a light microscope, and three areas with high expression were taken and photographed for storage (all pictures were taken at × 400).

### Western blot

Proteins of knee cartilage from each group were extracted and collected using RIPA Lysis Buffer (KGP250, China), mixed with 10 μl phosphatase inhibitor, 1 μl protease inhibitor, and 5 μl 100 mM PMSF. Protein concentrations of each group were determined by the BCA kit (KGA902, China). Then, electrophoresis was performed, blocking with 10% milk powder for 2 h, incubated with primary antibodies anti-iNOS (diluted 1:500 affinity AF0199), anti-COX2 (diluted 1:1000 UK Abcam plcab 179800), VEGF-A (diluted1:500, UK Abcam plc ab1316), VEGFR2(diluted1:1000, UK Abcam plc ab39638). After washing again, secondary antibodies were added and incubated for 2 h at room temperature. Image-J software was used to analyze the gray scale.

### Statistical analysis

The results were displayed as mean ± SD. All data were analyzed with SPSS 19.0statistical software. Differences among the three groups were analyzed by one-way analysis and 푃 < 0.05 indicated statistical significance.

## Results

### Histopathological changes in cartilage by HE and S-O staining

**Fig. 1:**
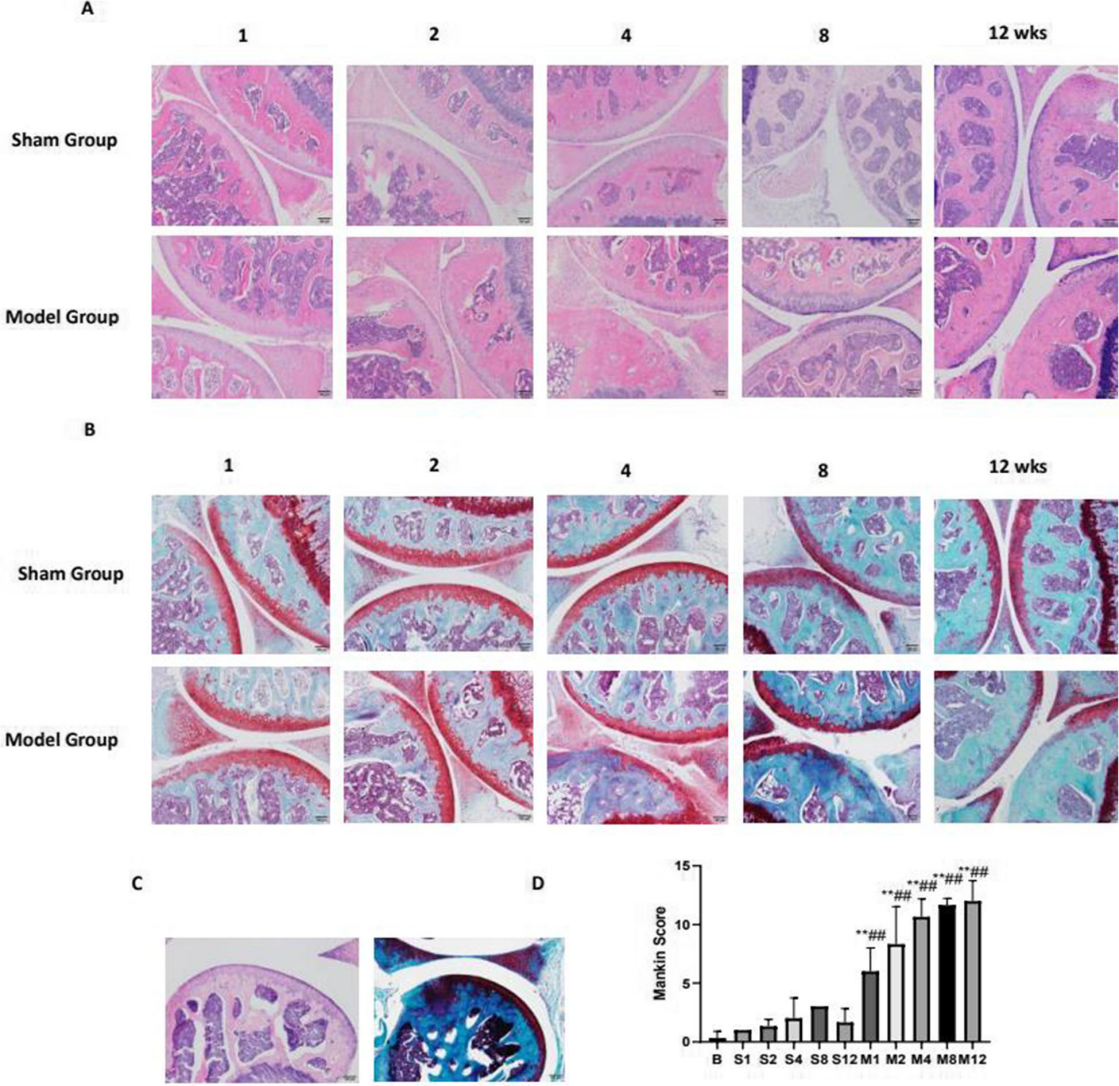
Histopathological analysis of the cartilage tissues obtained from each group (× 100) to show the development in the ACLT model. **A** After fixation, decalcification, and embedding, 4 mm frontal sections were cut from the knee joints and were stained with HE staining. **B** Safarin-O-fast green staining images of cartilage in each group. **C** HE and Safarin-O-fast green staining of the control group. **D** Mankin score. The data in the figures represent mean values ± SD, *p < 0.05, **p < 0.01 compared with the control group, #*P* < 0.05, ##*P* < 0.01 compared with the sham group at the same point

Hematoxylin-eosin (HE) and Safranin-O(S-O) staining were utilized to evaluate the changes of histological examination of cartilage, as shown in Fig. 1A, B. The HE and S-O staining showed that the cartilage surface was smooth and intact in the sham groups, similar to those of normal knee cartilage of mice in Fig.1C. However, the model groups exhibited cartilage superficial destruction, but limited to the superficial layers in 1 week. At 2 weeks, Safranin-O staining in the middle zone decreased. The defect of cartilage was developed to the calcified cartilage layer below the tidemark by 4 weeks and gradually extended to the full thickness of cartilage at 8 and 12 weeks. Next, we performed a modified Mankin score on the cartilage in different groups in Fig. 1D. The results showed that the Mankin scores of the experimental groups generally showed an increasing trend, and the difference between the model group and the sham group was statistically significant (*P* < 0.01). Indicating this surgery can lead to the degeneration of cartilage in mice. And the characteristics of the lesion were time-dependent.

### Changes of matrix biomarkers ADAMTS5, COL-II in knee joint cartilage

**Fig. 2:**
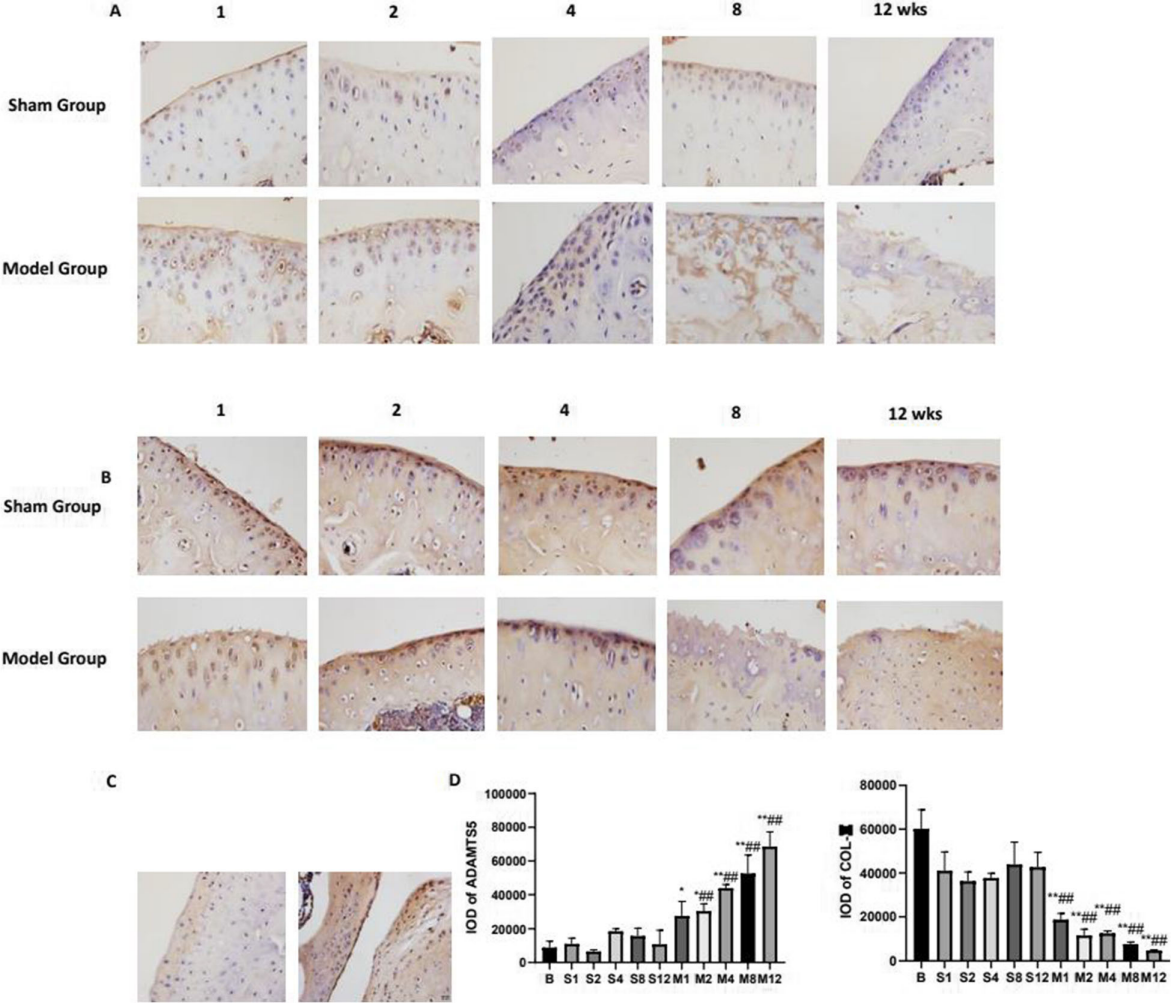
The expression of the cartilage matrix biomarkers ADAMTS5, COL-II of the knee joint. **A** Immunohistochemistry staining of ADAMTS5 (scale bar: 20 μm). **B** Immunohistochemistry staining of COL-II (scale bar: 20 μm). **C** The expression of cartilage matrix biomarkers ADAMTS5, COL-II in the blank group. **D** IOD of each group. The data in the figures represent mean values ± SD, *p < 0.05, **p < 0.01 compared with the control group, #P < 0.05, ##P < 0.01 compared with the sham group at the same point

Immunohistochemistry was used to detect the expression of ADAMTS5 and COL-II, both were important biomarkers to demonstrate ECM degradation of cartilage. The protein expression of ADAMTS5 in the sham groups showed weakly expressed. In the model groups, significant expression of ADAMTS5 was observed in the first week after surgery, and the location of positive cell extended to a deeper layer from 4 weeks. Because of the destruction of cartilage from 8 weeks, the chondrocytes were hardly to been seen, especially in 12 weeks, so it was hard to calculate the number of positive cells as shown in Fig. 2A. And the change was basically consistent with the histopathological results. COL-II was strongly stained in all zones of the articular cartilage both above and below the tidemark in sham groups. In the model groups, the expression of COL-II showed gradually decreased with the extension of experiment time and dramatically decreased from 4 weeks in Fig. 2B.

### The protein expression of inflammatory biomarkers COX-2 and iNOS in cartilage

**Fig. 3:**
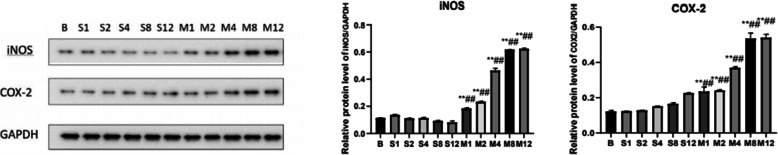
The protein level of inflammatory biomarkers COX-2 and iNOS in cartilage detected by western blotting. The data in the figures represent mean values ± SD, *p < 0.05, **p < 0.01 compared with the control group, #*P* < 0.05, ##*P* < 0.01 compared with the sham group at the same point

The expression of COX-2 and iNOS in the cartilage of each group was detected by western blot. As shown in Fig. 3A, B, the expression of COX-2 and iNOS gradually upregulated after ACLT surgery. The expression pattern was correlated with the degree of osteoarthritis. The results in this chapter indicate that inflammation in cartilage was involved in the development of osteoarthritis.

### Angiogenesis in the cartilage of ACLT-induced mouse

**Fig. 4:**
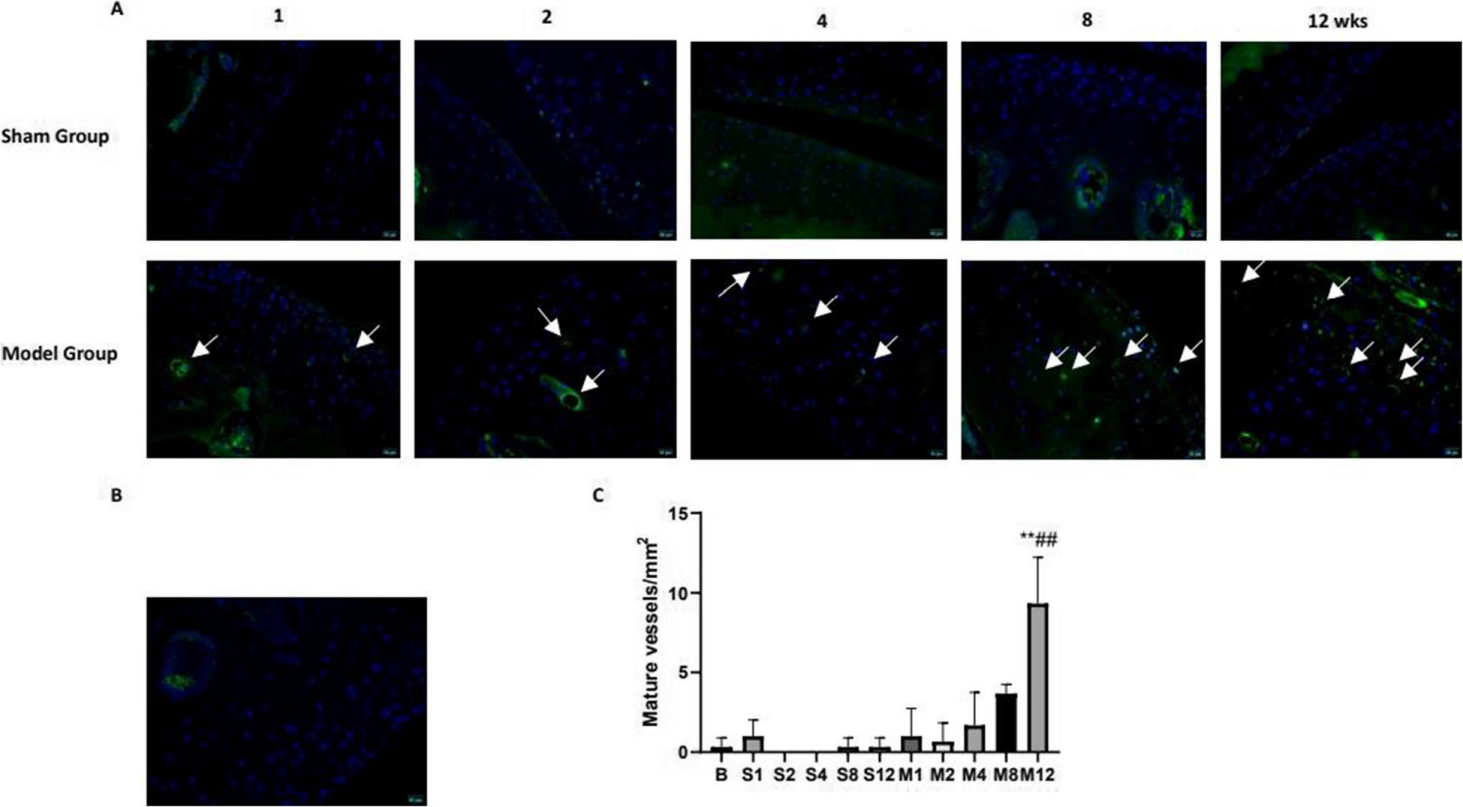
Immunofluorescence staining for CD31 to identify angiogenesis in the cartilage of ACLT-induced mouse by a fluorescence microscope (OLYMPUS) (scale bar: 20 μm). **A** CD31-positive staining in the cartilage of the sham operation group and the model group. **B** CD31-positive staining in the blank group. **C** Blood vessel count in each group. The data in the figures represent mean values ± SD, *p < 0.05, **p < 0.01 compared with the control group, #P < 0.05, ##P < 0.01 compared with the sham group at the same point

Immunostaining of CD31 was used to visualize the vascular invasion; as described in Fig. 4, CD31-positive cells in sham groups were rare, and new blood vessel was not found. In the cartilage of OA mice, a new blood vessel can be seen from 1 week at the osteochondral junction and cartilage. The number of blood vessels increased in 8 and 12 weeks. This indicated that vascular invasion was involved in the development of OA.

### The pattern of protein expression of VEGF-A and VEGFR2 in the articular cartilage

**Fig. 5:**
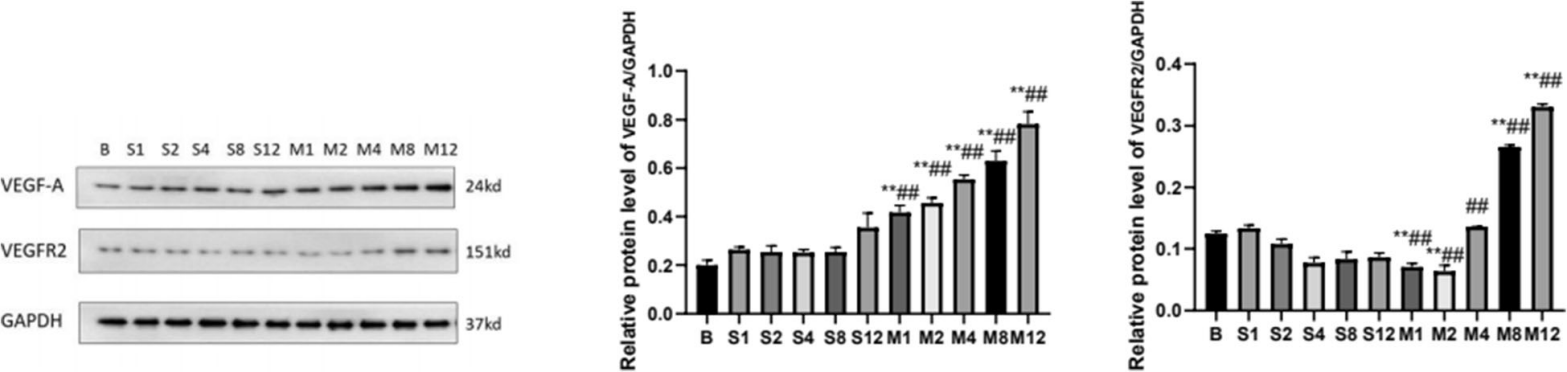
The level of the expression of VEGF-A and VEGFR2 in cartilage. The data in the figures represent mean values ± SD, *p < 0.05, **p < 0.01 compared with the control group, #*P* < 0.05, ##*P* < 0.01 compared with the sham group at the same point

To further explore the underlying mechanism of angiogenesis, we next investigated the protein level of VEGF-A and VEGFR2 in the cartilage at different pathological stages of OA with western blot (Fig. 5A, B).VEGF was a well-known angiogenic factor; according to our study, expression of VEGF-A was a particular feature in OA and demonstrated that VEGF-A and VEGFR2 were associated with vascular invasion at cartilage. The expression pattern of VEGF-A was basically consistent with the Mankin score. But the expression of VEGFR2 showed a decrease within 2 weeks and then increased from 4 weeks, significantly at 8 weeks in Fig. 5, which needs to be further studied.

## Discussion

In order to explore the pathogenesis of OA, animal models are often used as study subjects. Due to the rapid progress of mouse genomics and the availability of transgenic and knockout mice, the mouse is now the most ideal animal model for the study of molecular backgrounds of physiological and pathological conditions [7]. Among which, ACLT-induced OA mouse model, a stress-induced model, was often used in OA [7]. Here, we made a moderate OA model based on the description of Kamekura, it was described that the moderate model seemed suitable to reflect the entire process of OA including early-stage changes. After the surgery, HE and Safranin-O Fast Green staining were used to observe the pathological changes after surgery and make sure the model is successful. According to the histopathological analysis and Mankin scores, the results indicated that the severity of cartilage degeneration in the experiment group was closely related to the time after surgery. COL-II and aggrecan are considered as the most important structural components forming the normal Extracellular Matrix (ECM) in cartilage [17, 18]. The ECM maintains the balance due to the metabolism of chondrocytes, synoviocytes, and subchondral bone cells, and once the balance is broken, the cartilage was damaged, thus causing OA [19]. ADAMTS5, an important cartilage matrix-degrading enzyme, growing evidence showed it was involved in the pathogenesis of aggrecan cleavage of OA [20]. So we use COL-II and ADAMTS5 as matrix biomarkers in this research to demonstrate the damage of the cartilage. In this experiment, immunohistochemistry results showed that the expression of ADAMTS5 increased from 1 week, while collagen II decreased with the extension of experiment time.

The research of OA pathogenesis showed that normal articular cartilage is avascular but angiogenesis at the osteochondral junction and in non-calcified cartilage was observed in OA [21–23]. Loss of resistance to vascular invasion distinguishes OA cartilage from normal articular cartilage, which may be important in the pathogenesis of OA [24].CD31 is a marker of endothelial cells expressed in vascular development and is often used to identify newly formed blood vessels [25]. Here, we investigated the CD31 by immunostaining to visualize the vascular invasion. As shown, the blood vessels have not been observed in the sham group. In model groups, new blood vessels can be seen from 1 week and the number of blood vessels increased in 8 and 12 weeks, and this result was nearly correlated with the degree of osteoarthritis, which confirmed that angiogenesis played a significant role in the degeneration of cartilage.

The mechanism of angiogenesis involves a coordinated signaling axis, among which the VEGF played an important role. VEGF expression has been found to be increased in the articular cartilage, subchondral bone, synovium, synovial fluid, and serum of OA patients [26–30]. VEGF-A is the founding member of the VEGF family, and it binds to VEGF receptor-2 (VEGFR2) and plays the most important role [31]. Previous studies have confirmed that VEGF-mediated vascular invasion plays an important role in OA, which could lead to increased production of matrix metalloproteinase (MMP)1, MMP-3, and MMP-13, and induce chondrocyte apoptosis and inflammatory reaction as well as increased expression of nerve growth factor (NGF) [32–34]. Assessment of VEGF as a biomarker in patients with OA showed that increased VEGF in synovial fluid was correlated with the grade of OA severity [35]. Blocking VEGF signaling pathways and angiogenesis has emerged as a promising approach in recent preclinical studies in OA [36]. Our previous study has confirmed that Chinese medicine can decrease the VEGF expression and thus delay the progression of OA. But the change of VEGF-A/VEGFR2 expression in the ACLT-induced OA model was still unknown. In the current study, we found cartilage expression of VEGF-A was basically consistent with angiogenesis, which indicated VEGF-A was a particular feature in OA and more fully demonstrated that VEGF-A and VEGFR2 were associated with vascular invasion at cartilage.

Previous studies indicated that VEGF-A may have specific roles in inflammation, which are closely related to the processes of OA. Although OA is commonly described as a non-inflammatory disease in order to distinguish it from “inflammatory arthritis,” such as rheumatoid arthritis (RA) or the seronegative spondyloarthropathies [12], many studies showed that inflammation triggered by factors like biomechanical stress was involved in the development of OA [37]. Researches have confirmed that a variety of inflammatory mediators are involved in the pathological process of OA. COX-2 plays an important role in joint destructio n[38]; iNOS produces high levels of NO, which can inhibit the synthesis and secretion of ECM in chondrocytes, leading to cartilage degradation [39]. To confirm the inflammation reaction in this model, the expression of inflammatory biomarkers COX-2 and iNOS in cartilage were tested by western blot. Our results showed expression of COX-2 and iNOS increased in the model group, basically related to the Mankin scores and expression of VEGF-A.

## Conclusion

Taken together, we successfully established a surgical induced OA model with anterior cruciate ligament transection surgery (ACLT). The degenerative degree of articular cartilage was time-dependent, and angiogenesis and inflammation were important pathological changes of cartilage in KOA. The expression of VEGF-A/VEGFR2 signaling pathway was basically correlated with the degree of KOA. However, as far as we are concerned, the expression changes were tested in protein level, changes in gene level needed to be further investigated. In summary, these findings can provide experimental support for this model as a vector in the study of KOA pathogenesis

## Data Availability

The datasets used and/or analyzed during the current study are available from the corresponding author on reasonable request.

## Abbreviations

OA: Osteoarthritis
ACLT: Anterior cruciate ligament transection
VEGF: Vascular endothelial growth factor
VEGF-A: Vascular endothelial growth factor-A
VEGFR2: Vascular endothelial growth factor recepter 2
HE: Hematoxylin–eosin
S-O: Safranin-O
IHC: Immunohistochemistry
IF: Immunofluorescence
ADAMTS-5: A Disintegrin and Metalloproteinase with Thrombospondin Motifs 5
COL-II: Collagen II
COX2: Cyclooxygenase-2
iNOS: Inducible nitric oxide synthase
MMP: Matrix metalloproteinase
ECM: Extracellular matrix
NGF: Nerve growth factor
